# Inhibition of levodopa metabolism to dopamine by honokiol short-chain fatty acid derivatives may enhance therapeutic efficacy in Parkinson’s disease

**DOI:** 10.1038/s41598-025-05072-3

**Published:** 2025-06-06

**Authors:** Gang Cheng, Micael Hardy, Jimmy B. Feix, Balaraman Kalyanaraman

**Affiliations:** 1 Department of Biophysics, Medical College of Wisconsin, 8701 Watertown Plank Road, Milwaukee, WI 53226 USA; 2 Aix-Marseille Univ, CNRS, ICR, UMR 7273, 13013 Marseille, France

**Keywords:** Short-chain fatty acids, Levodopa, Dopamine, Microbiome, Polyphenol, Parkinson’s disease, Neurodegeneration, Parkinson's disease, Metabolomics, Microbial communities

## Abstract

This study investigates the antimicrobial properties of honokiol (HNK), a naturally occurring polyphenol, when conjugated with short-chain fatty acids (SCFAs) such as butyrate. We examined the effects of HNK-SCFA ester conjugates on *Enterococcus faecalis*, a gut bacterium that metabolizes levodopa, a drug used to manage Parkinson’s disease symptoms. Our findings indicate that HNK-SCFA-esters (e.g., HNK-acetate, HNK-propionate, HNK-butyrate, and HNK-hexanoate) inhibit *E. faecalis* growth in a dose-dependent manner, followed by a temporary recovery period during which levodopa remains intact and unmetabolized. Notably, HNK-SCFAs exhibit enhanced cellular permeability and are hydrolyzed within bacterial cells, releasing HNK and SCFAs. These results suggest that HNK-SCFAs may reversibly modulate the gut metabolism of levodopa to dopamine, potentially enhancing its therapeutic efficacy in treating Parkinson’s disease.

**Supplementary Information:**

The online version contains supplementary material available at 10.1038/s41598-025-05072-3.

## Introduction

**Fig. 1:**
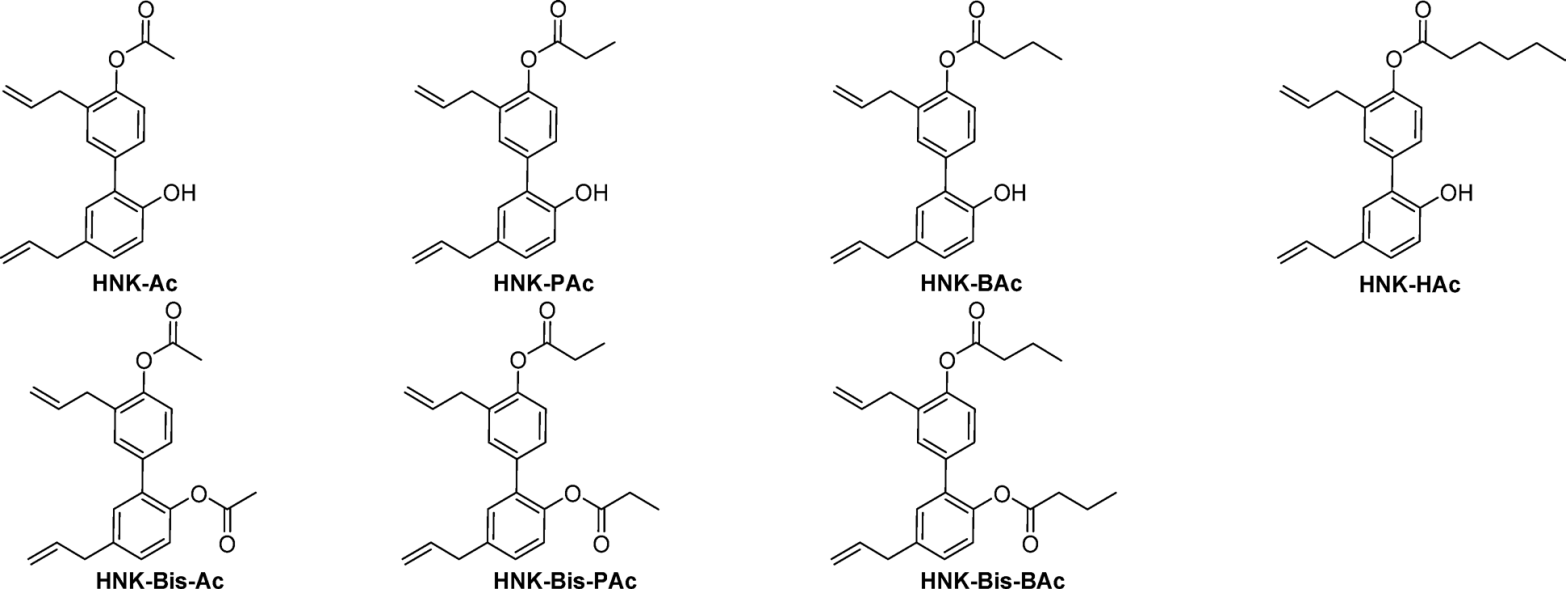
Chemical structures of HNK-SCFA-esters.

**Fig. 2:**
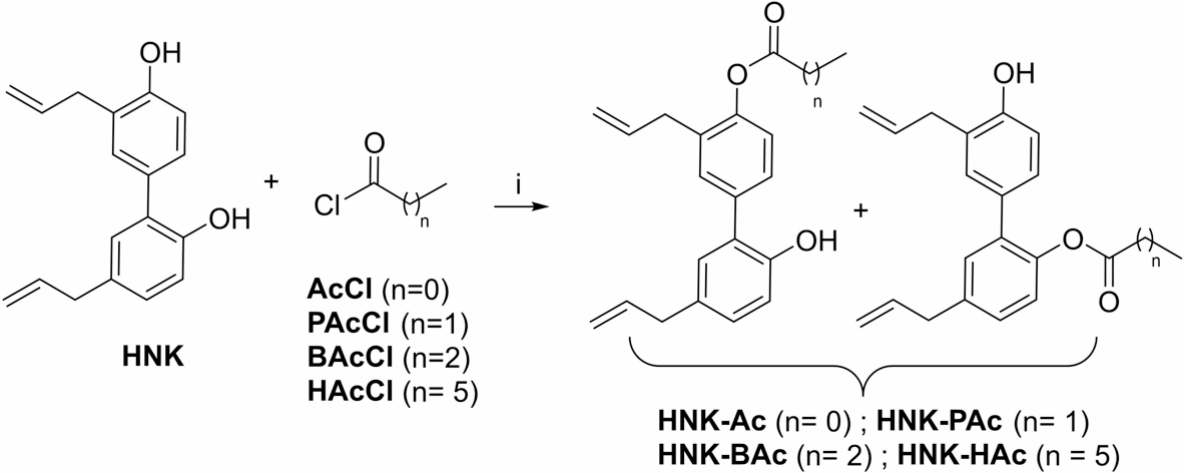
Syntheses of HNK-SCFA esters. Reagents and conditions used are: i, TEA, CH_2_Cl_2_, 24–45%.

Parkinson’s disease (PD) is a progressive neurodegenerative disorder characterized by motor dysfunction and non-motor symptoms. Honokiol (HNK), a naturally occurring polyphenol derived from the bark of the *Magnolia* genus, has demonstrated neuroprotective effects in several PD mouse models^1,2^. To enhance its therapeutic potential, we investigated a novel strategy involving the synthesis of HNK conjugates with short-chain fatty acids (SCFAs), generating a series of HNK-SCFA-esters (Figs. 1 and 2). These esters are designed to undergo hydrolysis by gut-derived esterases, thereby releasing both HNK and SCFAs in the gut.

Representative HNK-SCFA conjugates include honokiol acetic acid (HNK-Ac), propionic acid (HNK-PAc), butyric acid (HNK-BAc), and hexanoic acid (HNK-HAc), along with their *bis*-ester counterparts. The dual release of HNK and SCFAs offers the potential for synergistic neuroprotective effects. Although similar phenolic lipids have previously been explored as prodrugs of butyric acid for antibacterial applications^3^, and polyphenol-SCFA conjugates have been described^4^, their relevance to PD pathophysiology has not been established. Considering the unmet need for novel pharmacological approaches targeting both motor and non-motor PD symptoms, the development of HNK-SCFA esters represents a promising therapeutic strategy^5^.

Recent advances in microbiome research have highlighted the gut-brain axis as a key modulator of PD progression^6–10^. Patients with PD consistently exhibit decreased gut microbial diversity and diminished levels of SCFAs, particularly butyrate, due to decreased abundance of butyrate-producing bacteria^11–13^. This depletion correlates with the severity of both motor and non-motor symptoms, including depression in PD^13^. Moreover, sodium butyrate administration has been shown to restore striatal dopamine levels and improve motor performance in preclinical PD models^14^. Strategies aimed at augmenting gut-derived butyrate, through dietary supplementation, microbial modulation, or drug delivery, are of growing therapeutic interest in PD.

An additional microbiome-related challenge in PD is the compromised bioavailability of oral levodopa (L-dopa). Gut bacteria, particularly *Enterococcus faecalis*, metabolize L-dopa to dopamine via tyrosine decarboxylase in the gut, thereby reducing L-dopa’s systemic absorption and delivery to the brain^15–17^. Because dopamine produced in the gut cannot cross the blood–brain barrier, this microbial metabolism leads to decreased central dopaminergic activity. Importantly, deletion of the tyrosine decarboxylase gene in *E. faecalis* abolished L-dopa metabolism in the gut, underscoring its central role in this detrimental pathway^15–18^.

Although carbidopa, a peripheral aromatic L-amino acid decarboxylase inhibitor, is co-administered with L-dopa to prevent extracerebral metabolism, it does not inhibit bacterial tyrosine decarboxylases^15–17^. Consequently, gut microbial metabolism remains a barrier to therapeutic efficacy of L-dopa. In this study, we demonstrate that selected esterase-cleavable HNK-SCFA conjugates delay bacterial L-dopa metabolism and attenuate dopamine formation in a dose-dependent manner.

To our knowledge, this is the first study to describe polyphenol-based SCFA conjugates with esterase-labile and antimicrobial properties relevant to PD. These findings establish a mechanistic basis for a multifunctional therapy that integrates gut microbiome modulation, neuroprotection, and preservation of L-dopa bioavailability. This approach represents a significant step toward the development of gut-targeted pharmacological interventions for PD.

## Results

### Syntheses of HNK-SCFAs

HNK-SCFAs were synthesized by reacting HNK with the appropriate alkenoyl chloride in the presence of triethylamine in dichloromethane (CH_2_Cl_2_) (Fig. 2). The structures and purities of the products were confirmed by NMR analyses (Figs. S1 and S2).

### Esterase-induced hydrolysis of HNK-SCFAs

**Fig. 3:**
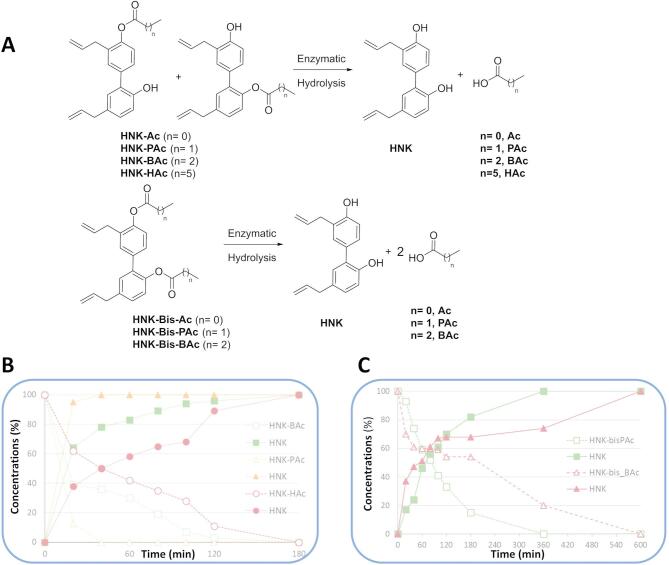
Kinetics of esterase-induced hydrolysis of HNK-SCFA-*mono* and *bis*-esters. (**A**) Schemes showing the hydrolysis of HNK-SCFA-esters and the *bis*-esters. (**B**) Kinetics of esterase-induced hydrolysis of HNK-SCFAs. HNK-SCFA-esters (100 µM) were incubated in the presence of esterase (30 U/mL). (**C**) Kinetics of esterase-induced hydrolysis of HNK-SCFA-*bis*-esters. HNK-SCFA-*bis*-esters (100 µM) were incubated in the presence of esterase (30 U/mL).

HNK-SCFA-esters (HNK-Ac, HNK-PAc, HNK-BAc, HNK-HAc, and corresponding *bis*-esters) were hydrolyzed by esterase enzymes. Figure 3 illustrates the time-dependent formation of HNK and the corresponding SCFA. Notably, no hydrolysis was observed in vitro when HNK-SCFAs were incubated in formate buffer (pH = 3) at 37 °C (data not shown). Results showed that HNK-Ac and HNK-*Bis*-Ac underwent esterase-induced hydrolysis very rapidly (Fig. S3). Hydrolysis rates decreased with increasing chain length in the order: HNK-PAc < HNK-BAc < HNK-HAc.

### Inhibition of *E. faecalis* proliferation by HNK-SCFAs

**Fig. 4:**
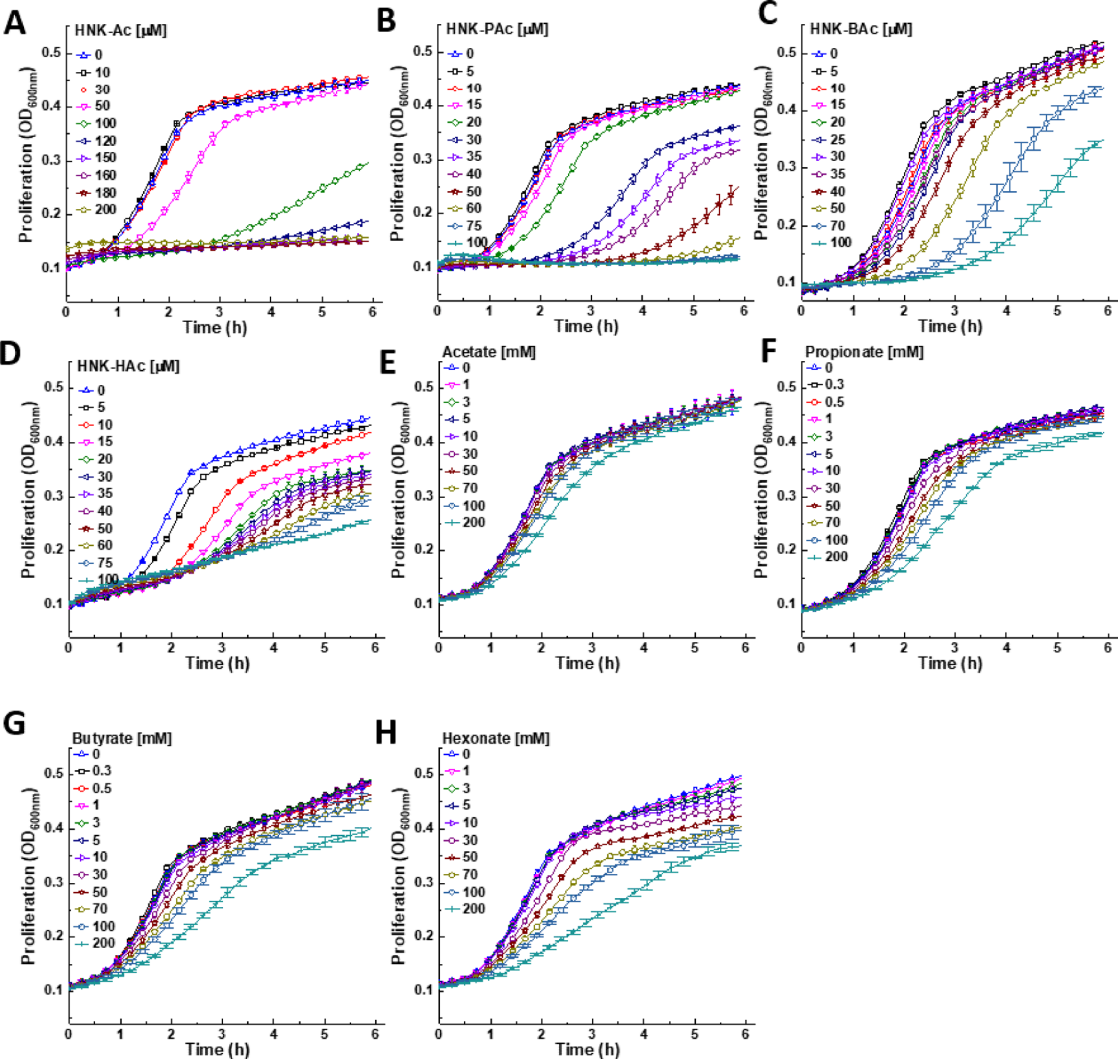
Effect of SCFAs and HNK-SCFA-esters on *E. faecalis* proliferation. The effects of HNK-Ac (**A**), HNK-PAc (**B**), HNK-BAc (**C**), HNK-HAc (**D**), acetate (**E**), propionate (**F**), butyrate (**G**), and hexaonate (**H**) and on the proliferation of *E. faecalis* were monitored at OD 600 nm for 6 h. Data shown are the mean ± SD, *n* = 4.

Unlike HNK (Fig. 4A), HNK-SCFAs bearing esterase-cleavable groups (e.g., HNK-BAc) induced a dose-dependent delay in *E. faecalis* proliferation (Fig. 4B, C, and D). After the time lag, bacterial growth resumed at a normal rate. In contrast, SCFAs alone inhibited *E. faecalis* proliferation without a time lag at millimolar levels (Fig. 4E–H).

### Uptake and hydrolysis of HNK-SCFA conjugates by *E. faecalis*

**Fig. 5:**
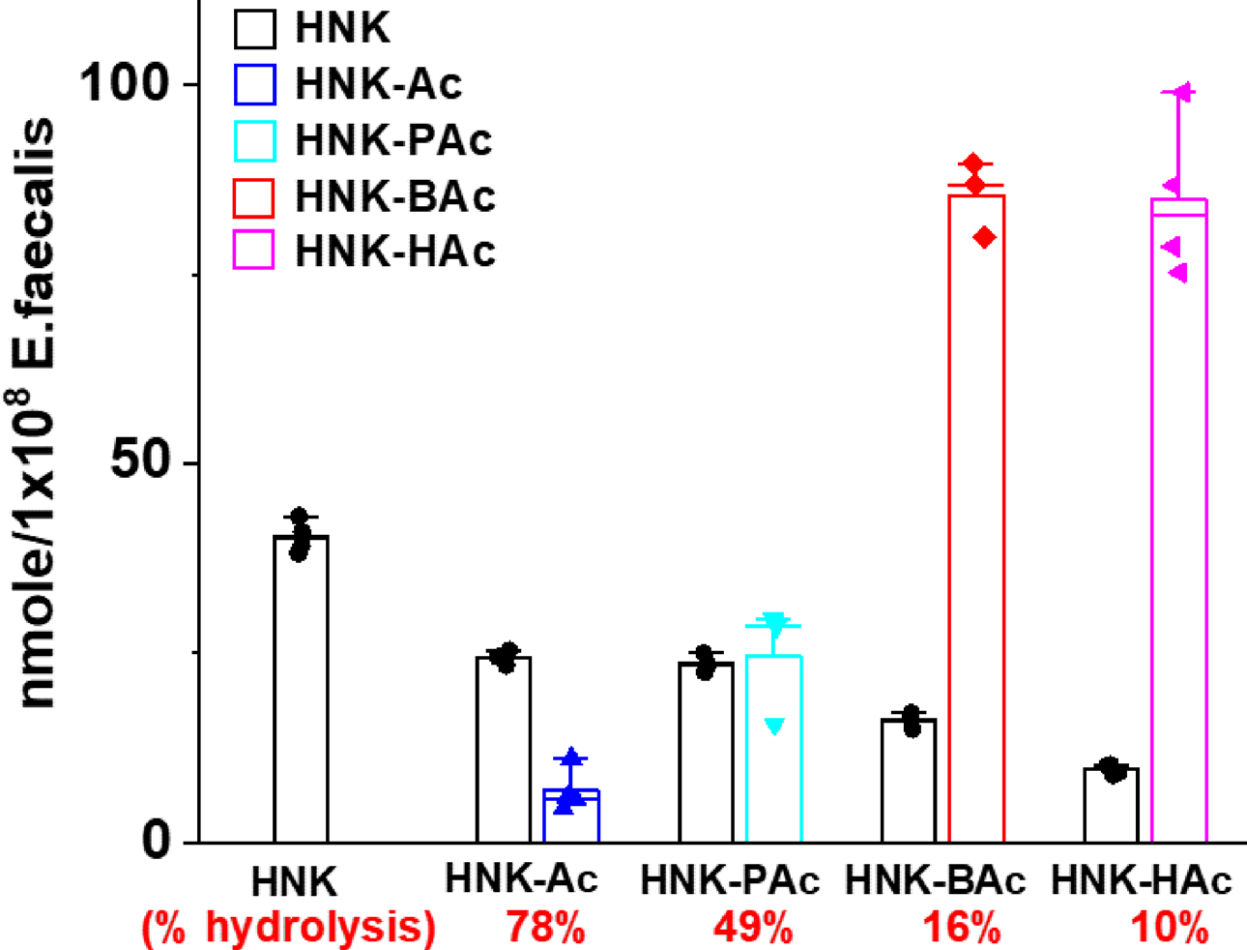
Uptake and hydrolysis of HNK SCFA analogs in *E. faecalis* cells. *E. faecalis* cells were treated with 50 µM of HNK, HNK-Ac, HNK-PAc HNK-BAc, or HNK-HAc for 1 h. The uptake and percent hydrolysis of these compounds in *E. faecalis* cells are shown for each analog.

Figure 5 illustrates the uptake and hydrolysis of HNK-Ac, HNK-PAc, HNK-BAc, and HNK-HAc by *E*. *faecalis* over 1 h. Intracellular concentrations increased with chain length (HNK-HAc = HNK-BAc > HNK-PAc > HNK-Ac) (Fig. S4). However, bacterial esterase-induced hydrolysis of HNK-SCFAs decreased with increasing chain length. Approximately 80% of HNK-Ac, 50% of HNK-PAc, 16% of HNK-BAc, and 10% of HNK-HAc were hydrolyzed to HNK. SCFAs (e.g., acetate, propionate, and butyrate) released from the hydrolysis of HNK-SCFA conjugates were not detectable using liquid chromatography–mass spectrometry (LC-MS)^19^, indicating the need for other mass spectrometry approaches such as GC-MS analyses.

### Effect of HNK-SCFAs on L-dopa metabolism in *E. faecalis* cells

**Fig. 6:**
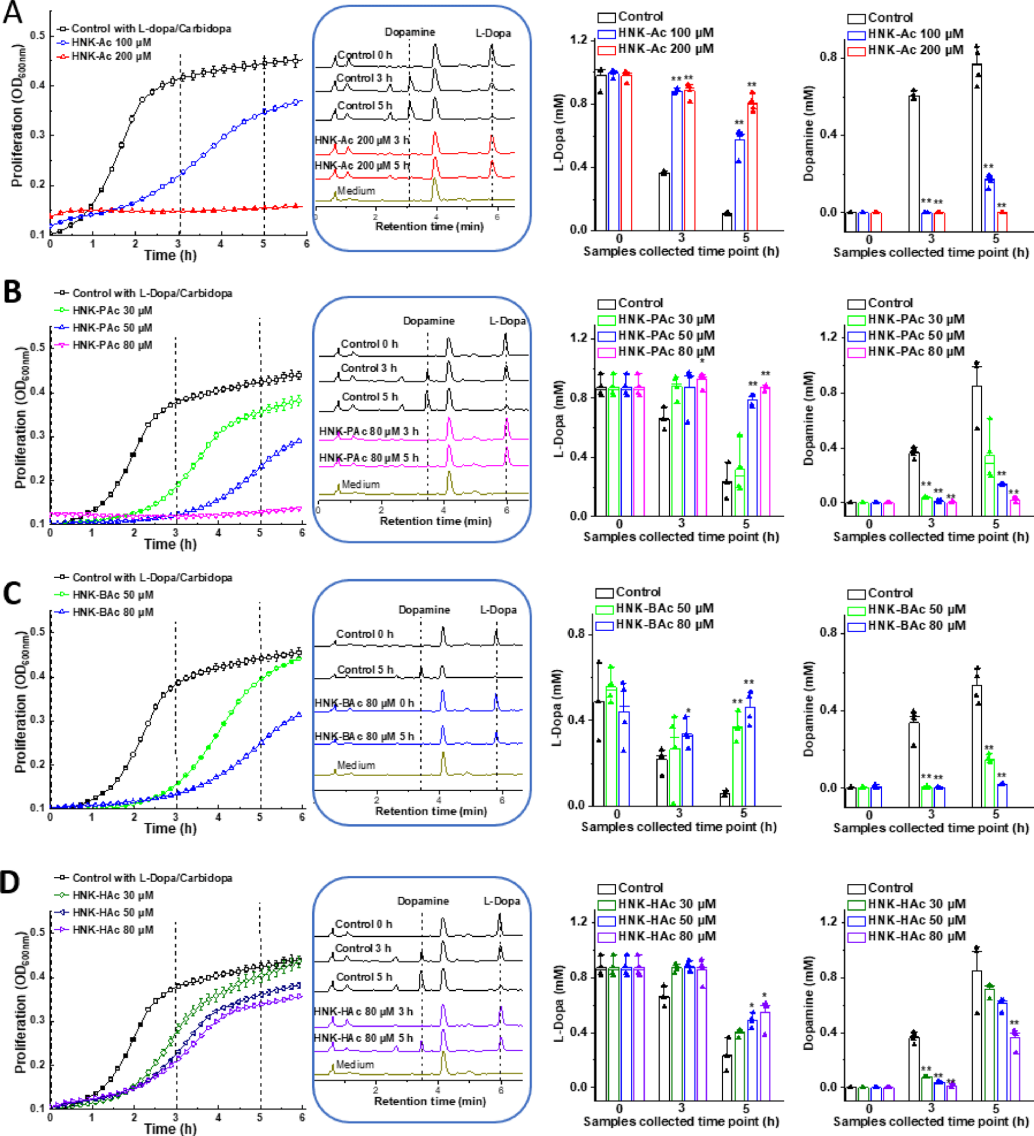
Effect of HNK-SCFA-esters on L-dopa degradation by *E. faecalis*. *E. faecalis* was treated with HNK-Ac (**A**), HNK-PAc (**B**), HNK-BAc (**C**), and HNK-HAc (**D**) as indicated, in the presence of 1 mM L-dopa and 0.22 mM of carbidopa. The effects of each analog (*left*), on the proliferation were monitored at OD600 for 6 h and culture media were collected at indicated time points (dashed lines) for L-dopa and dopamine measurements. HPLC traces of representative samples and standards are shown in the middle left panel. The effects on L-dopa consumption and dopamine formation are shown respectively. *, *P* < 0.05 vs. control group at each collection time point. Data shown are the mean ± SD, *n* = 4.

L-dopa is routinely used in combination with carbidopa in PD management. Therefore, we examined the effect of carbidopa (using the same L-dopa:carbidopa ratio as in clinical use) on L-dopa metabolism by *E. faecalis* treated with HNK-SCFAs. At clinically relevant ratios, carbidopa did not affect bacterial L-dopa metabolism, as it is a poor substrate for bacterial tyrosine decarboxylase^15–17^. Figure 6A–D shows that HNK-SCFAs dose-dependently decreased L-dopa degradation and dopamine formation by *E. faecalis.* Notably, both HNK-PAc and HNK-BAc inhibited dopamine formation more effectively than HNK-Ac. In contrast, HNK did not exhibit a similar pattern; with increasing doses, there was a total inhibition of *E. faecalis* proliferation (see Fig. 1 in Ref.^20^).

### Effect of HNK-SCFA-esters on membrane potential

**Fig. 7:**
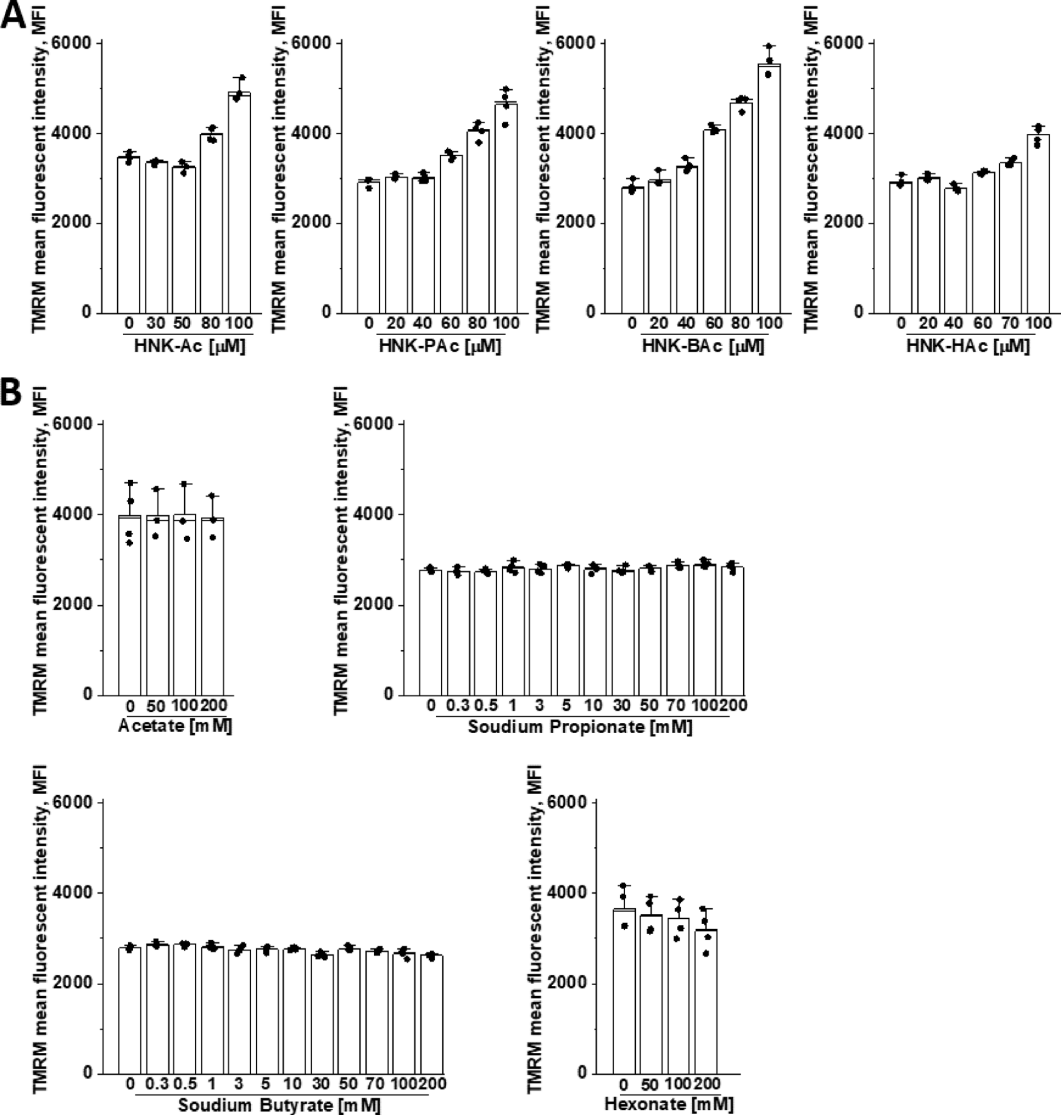
Effect of HNK-SCFA-esters on *E. faecalis* membrane potential. *E. faecalis* was treated with HNK, HNK-Ac, HNK-PAc, HNK-BAc, and HNK-HAc (**A**) and acetate, propionate, butyrate, and hexanoate (**B**) as indicated for 1 h. The effects on the membrane potential were measured by TMRM dye, the fluorescence indicator to determine the percentage change in TMRM fluorescence intensity between the control and treatments groups. The lower levels of TMRM fluorescence resulting from treatment reflect the depolarization of mitochondrial membrane potential. **, *P* < 0.01 vs. control group at each collection time point. Data shown are the mean ± SD, *n* = 4.

As shown in Fig. 7A, HNK-PAc, HNK-BAc, and HNK-HAc induced a dose-dependent increase in *E. faecalis* membrane potential, with HNK-BAc exhibiting the strongest effect among the HNK-SCFA conjugates. In contrast, acetate, propionate, and butyrate did not affect membrane potential across a range of concentrations (Fig. 7B). These findings are consistent with the observed enhanced uptake of HNK-SCFA-esters in the *E. faecalis* system (Fig. 6). The paradoxical effects of SCFAs in bacterial versus mammalian cells are further discussed in the “Discussion” Section.

### Effects of HNK-SCFAs on ATP levels in *E. faecalis*

**Fig. 8:**
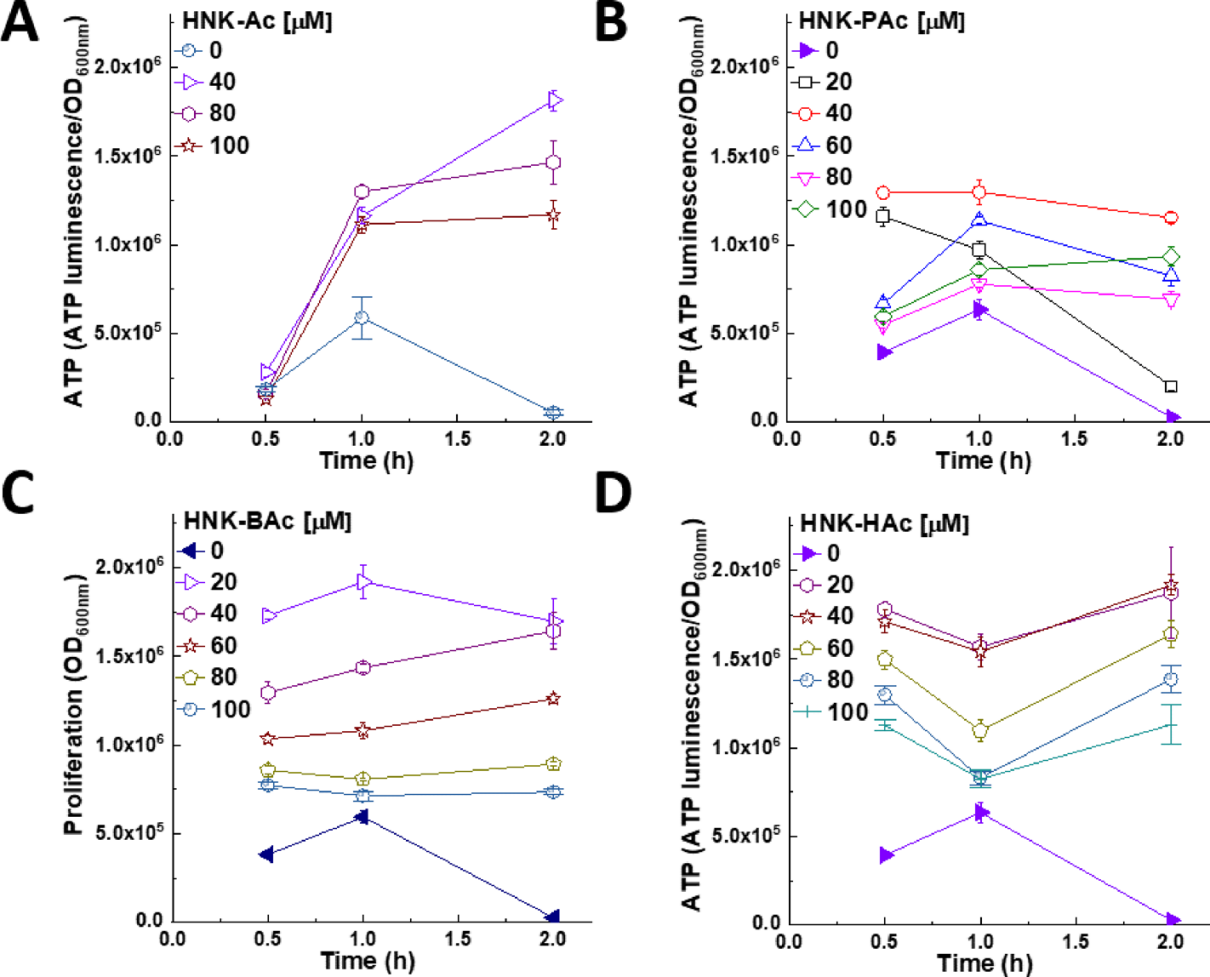
Effect of HNK-SCFA-esters on ATP production in *E. faecalis*. *E. faecalis* was treated with HNK-Ac (**A**), HNK-PAc (**B**), HNK-BAc (**C**), and HNK-HAc (**D**) as indicated for 0.5, 1, and 2 h.

Intracellular adenosine triphosphate (ATP) was measured in *E. faecalis* cells exposed to HNK-SCFAs and SCFAs. As shown, the effects of HNK-SCFAs on ATP formation varied (Fig. 8). Initially, HNK-SCFAs enhanced ATP levels followed by a decrease. SCFAs alone (Fig. S5) increased ATP levels in *E. faecalis* cells at millimolar concentrations.

HNK-BAc induced a dose- and time-dependent increase in ATP in the *E. faecalis* system. In HNK-BAc treated *E. faecalis*, ATP levels were initially higher in the presence of HNK-SCFAs, likely due to decreased ATP utilization under suppressed proliferation. After a dose-dependent time lag, the proliferation rates of *E. faecalis* increased over time in the presence of HNK-SCFAs. Accordingly, ATP levels began to decrease due to utilization (Fig. 8).

In the presence of SCFAs alone, ATP levels increased (Fig. S5). This increase is likely due to the utilization of SCFAs, such as acetate, by *E. faecalis* to generate more ATP. However, it is important to note that high millimolar concentrations of SCFAs were used in this experiment.

### Antimicrobial effects of HNK-SCFAs

Antimicrobial activity was assessed using a standard micro-dilution assay to determine minimal inhibitory concentration (MIC), the lowest concentration that completely inhibits bacterial growth during overnight incubation at 37 °C^21^. HNK-Ac had a MIC of 180 µM when tested against *E. faecalis*. None of the other HNK derivatives exhibited antimicrobial activity at the highest concentration tested (180 µM). This is consistent with results from proliferation assays indicating that these compounds inhibit bacterial growth for periods of up to 6 h, after which proliferation recovers.

### Cytotoxicity of HNK-SCFAs

Cytotoxicity was monitored in real time by SYTOX Green staining. The SYTOX measurements showed that HNK-SCFAs were non-cytotoxic at concentrations inhibiting > 90% proliferation of *E. faecalis* (Fig. S6). In contrast, the antibiotic ampicillin was cytotoxic at concentrations inhibiting *E. faecalis* proliferation^20^.

### Calculated values of the octanol/water partition coefficients

 The hydrophobicity of HNK-SCFAs was assessed by calculating the log P partition coefficients^22,23^. The calculated log P values for HNK, HNK-Ac, HNK-PAc, HNK-BAc, and HNK-HAc are 5.2, 5.1, 5.9, 6.3, and 7.2, respectively (Table S1). The log P values of HNK-ester analogs (*mono* and *bis*-esters) were assessed using a QSAR (quantitative structure–activity relationship) analysis and rational drug design as a measure of molecular hydrophobicity (Table S1). This method also uses a consensus model built using the ChemAxon software (San Diego, CA)^22,23^.

## Discussion

This study has significant scientific impact as it reveals a novel therapeutic potential to mitigate the gut metabolism of L-dopa to dopamine using nontoxic SCFA conjugates of HNK, a naturally occurring polyphenol. Both HNK and SCFAs have been shown to be nontoxic and neuroprotective. HNK is a commercially available nutritional supplement that is also widely used as a drug to induce sleep. SCFAs are key players in the interplay between diet, microbiota, and health^24,25^. SCFAs, including acetate (two carbons), propionate (three carbons), and butyrate (four carbons), are produced through anerobic fermentation of dietary fibers by the colonic microbiome^26^. The relative amounts of SCFA released in the gut depend on the type and amount of ingested fiber.

Propionate, a major microbial fermentation-induced metabolite in the human gut, has been shown to be neuroprotective in PD^27–30^. Additionally, butyrate-generating bacteria have been associated with neuroprotection in PD^31^. Increasing gut butyrate through prebiotic butyrogenic fibers has been proposed as a potential therapy^32^. The bioavailability of L-dopa in the brain decreases in patients with PD due to the increased metabolism of L-dopa to dopamine by gut bacteria, specifically *E. faecalis*^15–17^. The abundance of *E. faecalis* in human gut microbiota samples strongly correlates with L-dopa metabolism^15^, and patients with PD have varying levels of these bacteria. Thus, decreasing bacterial metabolism is a promising therapeutic approach to enhance the bioavailability of L-dopa in the brain. Previously, we showed that HNK, conjugated to a triphenylphosphonium moiety, mitigated the metabolism of L-dopa—alone or combined with carbidopa—to dopamine. Mito-*ortho*-HNK suppressed the growth of *E. faecalis*, decreased dopamine levels in the gut, and increased dopamine levels in the brain. Here, we show that mitigating the gut bacterial metabolism of L-dopa using a hybrid molecule consisting of a naturally occurring molecule and an endogenous gut metabolite could enhance the efficacy of L-dopa.

Results indicate that HNK-SCFAs enhanced the membrane potential of *E. faecalis*, resulting in hyperpolarization (Fig. 6A). Hyperpolarization results when the membrane potential becomes more negative, whereas depolarization occurs when the membrane potential becomes less negative or more positive. However, HNK and SCFAs alone did not affect the membrane potentials (Fig. 6B). The increase in membrane potential followed the order: HNK-BAc > HNK-PAc > HNK-HAc > HNK-Ac (Fig. 6A). This finding contradicts previous studies^33^. Under physiological pH values, the membrane potential of anaerobic gut commensal bacteria remained unchanged but slightly decreased at lower pH levels. In a model of colonic SCFA absorption using basolateral membrane vesicles from rat distal colonic mucosa, butyrate uptake was significantly higher at acidic extravesicular pH = 5.5 than at pH = 7.5^34^. Butyrate was reported to cause a reversible hyperpolarization in neurons due to increased intracellular calcium ions^35^. Propionate induced hyperpolarization in gallbladder epithelial cells^36,37^. Acetate-induced hyperpolarization was attributed differences in calcium permeability^38^.

HNK-SCFAs, as a novel class of prodrugs, have the potential to release two neuroprotective molecules: HNK and SCFAs like butyrate. Both molecules have been shown to inhibit neuroinflammation and reverse neurodegeneration^1,39–41^. HNK activates mitochondrial sirtuin-3 (Sirt-3), which induces antitumor and anti-inflammatory effects. Sirt-3 is implicated as a potential target for PD^42^.

HNK promotes mitophagy and mitochondrial dynamics through a Sirt-3-depenndent mechanism involving the AMPK-PGC-1alpha signaling pathway. Additionally, HNK activates the NAD^+^-consuming enzyme Sirt-3 to prevent neuron death and improve motor performance in a rat model of PD^43–46^.

HNK also decreases alpha-synuclein mRNA levels, potentially decreasing alpha-synuclein aggregation and the onset of neurological disorders collectively known as “synucleinopathies” including PD^47^. This study revealed a novel therapeutic target for modulating alpha-synuclein expression. Furthermore, propionate supplementation has been shown to reverse alpha-synuclein-induced neurodegeneration in *Caenorhabditis elegans*^48–50^. Enhancing SCFAs, such as propionate, in the gut through pharmacotherapy was shown to be beneficial in protecting against alpha-synuclein-induced neurodegeneration^50^. Therefore, HNK-SCFAs may provide a synergistic therapeutic effect in combating synucleinopathy. The therapeutic signaling of SCFA receptors has been proposed as a treatment for neuroinflammatory disorders^51^.

Although butyrate has been used as a nutritional supplement, its pungent and unfavorable odor has limited its widespread applications (Fig. S7). To overcome this problem, modified forms of butyrate were synthesized as prodrugs that release butyric acid through enzymatic hydrolysis (structures shown in Fig. S7). Proper administration of these drugs could offer advantages over probiotics. One such drug is arginine butyrate, an ester formed by combining arginine and butyrate, which is hydrolyzed to release arginine and butyrate in vivo^52^. Administering low doses of arginine butyrate restored membrane integrity and improved neuromuscular abnormalities in dystrophic mouse models^52^. These beneficial effects are attributed to HDAC (histone deacetylase) inhibition by butyrate and the inhibitory effects of nitric oxide synthase/arginine on intracellular calcium activity. Another prodrug, tributyrin (propane-1,2,3-triyl tributanoate), is a triglyceride derived from glycerol and three molecules of butyric acid. Present in butter and used in margarine, tributyrin serves as a postbiotic microbiome supplement. It is rapidly absorbed as a prodrug that is hydrolyzed by the lipase enzyme to butyric acid, inducing apoptosis and inhibiting prostate cancer cells^53^, and modulating gene transcription through HDAC inhibition^54^. Phenylalanine-butyramide, a novel butyrate derivative, has demonstrated protective effects against doxorubicin-induced cardiotoxicity^55^. It is also considered as a postbiotic that improves gut health by releasing butyrate. However, the mechanism of butyrate formation remains unclear. Additionally, amino acid-conjugated butyrate (e.g., serine-conjugated butyrate) has been used as a prodrug in autoimmune arthritis and neuroinflammation in preclinical mouse models^56^. Butyryl-L-carnitine, a butyrate ester of carnitine, is proposed as a prodrug for delivering carnitine and butyrate in the gut and preventing inflammation^57^.

Research suggests that L-dopa responsiveness in patients with PD may be associated with the abundance of the tyrosine decarboxylase gene in the gut^18^. Although this gene is found in bacteria like *Lactobacillus brevis* in humans, this gene is primarily associated with *E. faecalis*. Individuals with PD reportedly have decreased levels of SCFAs in their gut microbiome^58^. Additionally, bacteria that metabolize L-dopa in the small intestine have been detected in the feces of people with PD^59^. The metabolism of L-dopa by gut microbes and amino acid carboxylases in peripheral tissues contributes to the reduced availability of L-dopa in the brain.

A combination of L-dopa and carbidopa is the preferred treatment for managing PD symptoms. Although carbidopa does not prevent gut metabolism of L-dopa, it does inhibit peripheral metabolism of L-dopa by acting as a substrate inhibitor of peripheral amino carboxylases^15^. Interestingly, HNK-SCFAs may enhance the efficacy of L-dopa/carbidopa therapy by directly inhibiting both gut bacteria metabolism and peripheral metabolism of L-dopa^60^. Thus, a potential clinical implication of this work is the development of adjunctive pharmacomicrobiome therapy targeting the gut–brain axis^60^.

This study has some limitations. Notably, it did not demonstrate the enhanced therapeutic efficacy of L-dopa/carbidopa/HNK-SCFAs in a PD-relevant genetic model, such as the MitoPark mouse^61^. Additionally, the study did not investigate the effects of HNK-SCFAs on L-dopa-induced dyskinesia in a mouse model^62^.

A genetically engineered mouse model, the MitoPark mouse, recapitulates many of the phenotypic features (mitochondrial dysfunction, microglial activation, dopaminergic degeneration, dopamine deficiency, and progressive neuronal deficits and protein occlusion) of PD. We propose that the benefits of treating human PD with L-dopa can be potentiated by the use of adjunctive treatments utilizing HNK-SCFAs that inhibit the breakdown of L-dopa to dopamine in the gastrointestinal tract, thereby enhancing L-dopa conversion to dopamine in the brain. The MitoPark transgenic mouse model is particularly relevant, as it replicates key PD features including gastrointestinal dysfunction associated with PD^63^. In addition, it will be important to determine if the beneficial effects of HNK-SCFAs observed in the current study are maintained with repeated treatments over an extended period of time. Collaboratively, we have previously published the neuroprotective effects of mitochondria-targeted drugs in the MitoPark mouse^64,65^. Studies using MitoPark mice are not feasible at this time; however, future collaborative research will investigate these aspects.

## Methods

### Bacterial strain and culture conditions

*E. faecalis* (Cat# OG1RF) was obtained from the American Type Culture Collection (ATCC). *E. faecalis* was cultured and grown overnight in TSB (tryptic soy broth), diluted 1:200 into fresh TSB and then grown at 37 °C in flasks on a rotating shaker at 250 rpm to reach the exponential growth phase (optical density at 600 nm [OD_600_] of 0.2–0.5) before use in the in vitro assays.

### Synthesis and purification of HNK-SCFA conjugates

All reagents and solvents were purchased from commercial sources and used without further purification. HNK-SCFA esters were synthesized by acylation of HNK with the corresponding alkenoyl chlorides in CH₂Cl₂ in the presence of triethylamine. Reaction progress was monitored by thin layer chromatography using silica gel Merck ^60^F254. Crude materials were purified by flash chromatography on Merck Silica gel 60 (0.040–0.063 mm). ^1^H NMR spectra were acquired on a Bruker DPX AVANCE 400 spectrometer equipped with a quattro nucleus probe. ^1^H NMR and ^13^C were taken in deuterated chloroform (CDCl_3_) using CDCl_3_ and tetramethyl silane as internal reference respectively. Chemical shifts (δ) are reported in ppm and *J* values in Hertz (see Supplemental Materials section).

### Measurement of bacterial cell proliferation

For all proliferation assays, cells were diluted to the final OD_600_ of 0.1 with indicated treatments in a 96-well plate. Cell proliferation, which was represented as absorbance at 600 nm, was acquired in real time every 3 min for 6 h using a plate reader (BMG Labtech, Inc., Ortenberg, Germany) equipped with an atmosphere controller set at 37 °C, 100% air.

### Measurement of bacterial membrane potential

Membrane potential was measured using the fluorescence dye tetramethylrhodamine methyl ester (TMRM)^66–68^. Briefly, bacteria in the exponential growth phase (OD_600_ of 0.4) were treated with test compounds as previously indicated for the MTDs or commonly used antibiotics in a black, clear-bottom 96-well plate; then, an aliquot of TMRM was added at a final concentration of 50 nM for 20 min^15,66,68,69^. After incubation with TMRM, the plate was centrifuged twice at 2500 g for 5 min and washed with phosphate buffered saline. Fluorescence was monitored at an excitation of 544 nm and emission of 590 nm using a plate reader (BMG Labtech, Inc., Cary, NC). Data were collected as the mean fluorescent intensity and were normalized to the total OD_600_ as the total bacteria number. The effects on membrane potential were compared for potential correlation with the MIC and minimum bactericidal concentration values.

### Uptake and intracellular hydrolysis of HNK-SCFAs

*E. faecalis* cells in the exponential growth phase (OD_600_ = 0.3–0.5) were diluted to the final OD_600_ of 0.1 at 20 mL volume, then treated with HNK or HNK-SCFAs as indicated for 1 h. Cell pallets were collected by centrifugation at 2,500 g × 5 min at 4 °C and stored at − 80 °C before extraction was performed. The cell pallet was dissolved in dimethylsulfoxide (100 µL) and taken for high-performance liquid chromatography (HPLC) analysis.

HNK-SCFAs and HNK formed from hydrolysis were separated and monitored by HPLC using an Agilent 1200 apparatus equipped with ultraviolet-visible absorption. Typically, 4 µL of a sample was injected on a Phenomenex reverse phase column (Kinetex 2.6 µ, 100 mm × 4.6 mm). The absorption traces were collected at 254 nm. The compounds were separated by a linear increase in acetonitrile phase concentration from 10 to 100% over 14 min and until 17 min at 100% acetonitrile containing 0.1% (v/v) trifluoroacetic acid. The flow rate used was 1.3 mL/min.

### Measurement of intracellular ATP

Intracellular ATP was quantified using a luciferase-based ATP determination kit, according to the manufacturer’s instructions (Sigma Aldrich, St. Louis, MO, Cat# FLLAA). Following cell lysis, a luciferase/luciferin agent (Cat# FLAAM) was added to the cell lysates. After swirling, luminescence was measured using a luminometer, and results were normalized to total cell numbers, which was represented as absorbance at 600 nm (OD_600nm_).

### Cytotoxicity measurements

Cytotoxicity was evaluated using the SYTOX Green (Invitrogen, Cat# S7020)^20^. The SYTOX method labels the nuclei of dead cells, yielding green fluorescence. Fluorescence (Ex: 485 nm, Em: 535 nm) from the dead cells in the 96-well plate were recorded every 5 min for 3 h using a plate reader (BMG Labtech, Inc.) equipped with an atmosphere controller set at 37 °C.

*E. faecalis* cells in the exponential growth phase (OD_600_ = 0.4) were treated with HNK-SCFAs in a black, clear-bottom 96-well plate for 3 h, and dead cells were monitored in the presence of 200 nM SYTOX Green. Cell lysis reagent (B-PER complete bacterial protein extraction reagent, Thermo Scientific, Cat# 89821) was used as a positive control.

### L-dopa metabolism and LC-MS analysis

*E. faecalis* cells in the exponential growth phase (OD_600_ of ~ 0.4) were diluted to a final OD_600_ of 0.1 and treated with L-dopa (1 mM) alone or in combination with carbidopa (0.22 mM) as previously described^20^. At the indicated time points (1–6 h), samples (1 mL of media) were collected by centrifugation at 2,500 g × 5 min at 4 °C, and the supernatant was lyophilized. The dry residue consisting of L-dopa and metabolites was resuspended in ice-cold methanol (100 µL) and analyzed by LC-MS using an Agilent 1200 apparatus equipped with ultraviolet-visible absorption and a mass spectrometry detector (single quadrupole). Typically, 2 µL of a sample was injected on an Agilent Poroshell column (120 HILIC-Z, PEEK, 100 mm × 2.1 mm, 2.7 μm, 25 °C), with absorbance monitored at 280 nm.

### Esterase-mediated hydrolysis of HNK-SCFAs

HNK-SCFA-esters (1 mM) solutions were prepared in 100 mM phosphate buffer (pH = 7.4). Then, HNK-SCFA-esters (100 µM) were incubated with esterase (30 U/mL) (Sigma Aldrich, St. Louis, MO). A 4 µL mixture was injected into HPLC to monitor the cleavage of the esters with the release of HNK and the corresponding SCFAs.

HPLC analyses were performed using an Agilent 1200 system equipped with absorption detectors. The samples (5 µL) were injected into a reverse phase column (Phenomenex, Kinetex C18, 100 mm × 4.6 mm, 2.6 μm) equilibrated with 10% (v/v) MeCN, 90% (v/v) water containing 0.1% (v/v) trifluoroacetic acid.

HNK esters derivatives were eluted by increasing the content of MeCN (v/v) from 20 to 100% over 14 min and until 17 min at 100% acetonitrile at a flow rate of 1.3 mL/min. The absorption used to monitor the ester cleavages was 254 nm.

### Statistical analysis

All data were expressed as mean ± standard deviation (SD) or mean ± standard error of the mean (SEM), as indicated. Comparisons between treatment and control groups were performed using an unpaired Student’s t-test analysis. A p-value of less than 0.05 was considered statistically significant. Sample sizes (n) are indicated in figure legends.

## Electronic supplementary material

Below is the link to the electronic supplementary material.

Supplementary Material 1

## Data Availability

This study did not generate/analyze any computational datasets/code or publicly archived datasets. All data are provided within the manuscript. Requests for information, resources, and reagents are available from the corresponding author upon reasonable request.
