# Supplementary material for: Inhibition of levodopa metabolism to dopamine by honokiol short-chain fatty acid derivatives may enhance therapeutic efficacy in Parkinson’s disease
Source: Sci Rep. 2025 Jun 6;15:20004. doi: 10.1038/s41598-025-05072-3 (PMC12144245; doi:10.1038/s41598-025-05072-3)
Supplement: Supplementary file 1 — Supplementary Material 1 [file 41598_2025_5072_MOESM1_ESM.docx]

**Inhibition of levodopa metabolism to dopamine by honokiol short-chain fatty acid derivatives may enhance therapeutic efficacy in Parkinson’s disease**

Gang Cheng,^a^ Micael Hardy,^b^ Jimmy B. Feix,^a^ Balaraman Kalyanaraman^a,*^

^a^Department of Biophysics, 8701 Watertown Plank Road, Milwaukee, WI 53226, United States

^b^Aix-Marseille Univ, CNRS, ICR, UMR 7273, Marseille 13013, France

*Corresponding author: Balaraman Kalyanaraman, Department of Biophysics, Medical College of Wisconsin, 8701 Watertown Plank Road, Milwaukee, WI 53226, United States; balarama@mcw.edu; 414-955-4000

**Supplementary Materials**

**1. Overview**

Syntheses of honokiol acetic acid (HNK-Ac), honokiol *bis* acetic acid (HNK-*Bis*-Ac), honokiol propionic acid (HNK-PAc), honokiol *bis* propionic acid (HNK-*Bis*-PAc), honokiol butyric acid (HNK-BAc), honokiol *bis* butyric acid (HNK-*Bis*-BAc), and honokiol hexanoic acid (HNK-HAc) are presented in the following sections. Their nuclear magnetic resonance (NMR) results are presented in Figure S1 and Figure S2.

Chemical structures for butyrate derivatives are shown in Figure S3.

***2. HNK-Ac*** *(3',5-diallyl-2'-hydroxy-[1,1'-biphenyl]-4-yl acetate)*

***2.1 Synthesis***

HNK-Ac was prepared by reacting HNK (0.4 g, 1.5 mmol) in dichloromethane (CH_2_Cl_2_) (10 mL) with acetyl chloride (107 µL, 1.5 mmol) in the presence of triethylamine (217 µL, 1.5 mmol). The mixture was stirred at room temperature for 2 h. Then, water was added to the mixture and the product was extracted by diethyl ether (Et_2_O). The organic layer was dried over sodium sulfate (Na_2_SO_4_), and the solvent was removed under reduced pressure. Purification by flash chromatography (AcOEt/pentane, 85/15) delivered the corresponding HNK-Ac (115 mg, 25% yield) as a mixture of both isomers. HRMS calculated for HNK-Ac C_20_H_20_O_3_ [MNa]^+^ 331.1305, found, 331.1306.

***2.2 NMR Results***

^1^H NMR (400.13 MHz, CDCl_3_) δ, 7.38-7.33 (2H, m), 7.18-7.14 (1H, m), 7.11-7.07 (1H, m), 7.06-7.04 (1H, m), 6.92 (1H, d, *J* = 8.3), 6.06-5.84 (2H, m), 5.16-5.02 (5H, m), 3.40-3.33 (4H, m), 2.35 (3H, s. ^13^C NMR (75 MHz, CDCl_3_) δ, 169.4, 150.8, 148.6, 137.6, 135.5, 135.2, 132.9, 132.3, 131.1, 130.3, 129.3, 128.2, 127.2, 123.2, 116.6, 115.9, 115.6, 39.3, 34.7, 20.9.

**3. HNK-PAc** (3,5'-diallyl-2'-hydroxy-[1,1'-biphenyl]-4-yl propionate) **and HNK-*Bis*-PAc** (3',5-diallyl-[1,1'-biphenyl]-2,4'-diyl dipropionate)

***3.1 Synthesis***

To a mixture of HNK (0.25 g, 0.93 mmol) in CH_2_Cl_2_ (4 mL) was added propionyl chloride (82 µL, 0.93 mmol) in the presence of triethylamine (200 µL, 0.97 mmol). The mixture was stirred at room temperature for 2 h. Then, water was added to the mixture and the product was extracted by Et_2_O. The organic layer was dried over Na_2_SO_4_, and the solvent was removed under reduced pressure. Purification by flash chromatography (Et_2_O/pentane, 85/15) delivered the corresponding HNK-PAc (136 mg, 45% yield) and honokiol *para* propionic acid (HNK-*para*-PAc) (30 mg, 10%). HRMS calculated for HNK-PAc C_21_H_22_O_3_ [MH]^+^ 340.1907, found, 340.1909. HRMS calculated for HNK-*Bis*-PAc C_24_H_26_O_4_ [MH]^+^ 396.2169, found, 396.2171.

***3.2 NMR Results***

^1^H NMR (400.13 MHz, CDCl_3_) δ, 7.37-7.33 (2H, m), 7.18-7.13 (1H, m), 7.11-7.03 (2H, m), 6.91 (1H, d, *J* = 8.3), 6.07-5.84 (2H, m), 5.02-5.04 (5H, m), 3.40-3.33 (4H, m), 2.65 (2H, 2, *J* = 7.6), 1.31 (3H, 2t, *J* = 7.6). ^13^C NMR (75 MHz, CDCl_3_) δ, 172.9, 150.8, 148.6, 137.7, 135.6, 135.0, 132.9, 132.3, 131.1, 130.2, 129.2, 128.1, 127.3, 123.1, 116.6, 115.9, 115.6, 39.4, 35.1, 34.7, 27.7, 9.4.

**4. HNK-BAc** (3,5'-diallyl-2'-hydroxy-[1,1'-biphenyl]-4-yl butyrate [HNK-*ortho*-BAc])

***4.1 Synthesis***

To a mixture of HNK (0.25 g, 0.33 mmol) in CH_2_Cl_2_ (4 mL) was added butyryl chloride (137 µL, 1 mmol) in the presence of triethylamine (200 ml, 0.97 mmol). The mixture was stirred at room temperature for 2 h. Then, water was added to the mixture and the product was extracted by Et_2_O. The organic layer was dried over Na_2_SO_4_, and the solvent was removed under reduced pressure. Purification by flash chromatography (Et_2_O/pentane, 85/15) delivered the corresponding HNK-BAc (101 mg, 45% yield) and honokiol *para* butyric acid (HNK-*para*-BAc) (30 mg, 13%). HRMS calculated for HNK-BAc C_22_H_24_O_3_ [M+NH_4_]^+^, 354.2064, found, 354.2062.

***4.2 NMR Results***

^1^H NMR (400.13 MHz, CDCl_3_),δ 7.36-7.34 (2H, m), 7.15 (1H, d, *J* = 8.1), 7.01-7.04 (2H, m), 6.91 (1H, d, *J* = 8.1), 6.01-5.86 (2H, m), 5.22-5.02 (5H, m), 3.37 (4H, dd, *J* = 1, 6.6), 2.60 (2H, t, *J* = 7.3), 1.83 (2H, sext., *J* = 7.5, 14.8), 1.09 (3H, t, *J* = 7.3). ^13^C NMR (75 MHz, CDCl_3_) δ 172.0, 172.7, 150.8, 148.6, 137.7, 135.5, 135.1, 132.9, 132.3, 131.1, 130.2, 129.2, 128.1, 127.3, 123.1, 116.6, 115.9, 115.6, 39.3, 36.2, 34.6, 18.2, 13.7.

**5. HNK-HAc** (3,5'-diallyl-2'-hydroxy-[1,1'-biphenyl]-4-yl hexanoate [HNK-*ortho*-HAc] and 3',5-diallyl-4'-hydroxy-[1,1'-biphenyl]-2-yl hexanoate [HNK-*para*-HAc])

***5.1 Synthesis***

HNK-HAc was prepared by reacting HNK (0.35 g, 1.3 mmol) in CH_2_Cl_2_ (4 mL) with hexanoyl chloride (186 µL, 1.3 mmol) in the presence of triethylamine (200 µL, 0.97 mmol). The mixture was stirred at room temperature for 2 h. Then, water was added to the mixture and the product was extracted by Et_2_O. The organic layer was dried over Na_2_SO_4_, and the solvent was removed under reduced pressure. Purification by flash chromatography (Et_2_O/pentane, 85/15) delivered the corresponding HNK-HAc (115 mg, 24% yield) as a mixture of both isomers. HRMS calculated for HNK-HAc C_24_H_28_O_3_ [MNa]^+^ 387.1931, found, 387.1931.

***5.2 NMR Results***

^1^H NMR (400.13 MHz, CDCl_3_), δ 7.37-7.33 (1H, m), 7.23-6.77 (5H, m), 6.10-5.86 (2H, m), 5.22-4.99 (5H, m), 3.47-3.31 (4H, m), 2.61&2.35 (2H, 2t, *J* = 7.6, 7.3), 1.80 & 1.56 (2H, 2sext., *J* = 7.6, 14.9), 1.46-1.38 (2H, m), 1.32-1.18 (2H, m), 0.96& 0.88 (3H, 2t, *J* = 7.1, 6.9). ^13^C NMR (75 MHz, CDCl_3_) δ 172.3, 172.2, 153.6, 150.7, 148.6, 146.1, 137.9, 137.6, 137.1, 136.2, 135.4, 134.9, 134.4, 132.9, 132.3, 131.1, 131.0, 130.8, 130.2, 129.2, 128.1, 127.3, 123.2, 122.7, 116.7, 116.6, 116.1, 115.9, 115.6, 39.7, 39.4, 35.1, 34.6, 34.3, 34.2, 31.3, 31.2, 24.6, 24.4, 22.3, 22.2, 13.9, 13.8.

***6. HNK-Bis-Ac*** (3',5-diallyl-[1,1'-biphenyl]-2,4'-diyl diacetate)

***6.1 Synthesis***

HNK-Bis-Ac was prepared by reacting HNK (0.15 g, 0.56 mmol) in dichloromethane (CH_2_Cl_2_) (6 mL) with acetyl chloride (107 µL, 1.5 mmol) in the presence of triethylamine (217 µL, 1.5 mmol). The mixture was stirred at room temperature for 2 h. Then, water was added to the mixture and the product was extracted by diethyl ether (Et_2_O). The organic layer was dried over sodium sulfate (Na_2_SO_4_), and the solvent was removed under reduced pressure. Purification by flash chromatography (AcOEt/pentane, 85/15) delivered the corresponding HNK-*Bis*-Ac (160 mg, 81 % yield). HRMS calculated for HNK-Bis-Ac C_22_H_22_O_4_ [MNa]^+^ 373.1410, found, 373.1405.

***6.2 NMR Results***

^1^H NMR (400.13 MHz, CDCl_3_), δ 7.27-7.22 (2H, m), 7.20-7.14 (2H, m), 7.08-7.02 (2H, m), 6.01-5.82 (2H, m), 5.13-5.03 (4H, m), 3.40 (2H, d, *J* = 6.9), 3.30 (2H, d, *J* = 6.6), 2.30 (3H, s), 2.06 (3H, s). ^13^C NMR (75 MHz, CDCl_3_) δ 169.5, 169.3, 148.3, 145.9, 138.2, 136.9, 135.7, 135.5, 133.7, 131.6, 130.9, 130.8, 128.6, 127.9, 122.7, 122.3, 116.4, 116.3, 39.6, 34.7, 20.9, 20.8.

**7. HNK-*Bis*-PAc** (3',5-diallyl-[1,1'-biphenyl]-2,4'-diyl dipropionate)

***7.1 Synthesis***

To a mixture of HNK (0.15 g, 0.56 mmol) in CH_2_Cl_2_ (5 mL) was added propionyl chloride (82 µL, 0.93 mmol) in the presence of triethylamine (300 µL, 1.45 mmol). The mixture was stirred at room temperature for 2 h. Then, water was added to the mixture and the product was extracted by Et_2_O. The organic layer was dried over Na_2_SO_4_, and the solvent was removed under reduced pressure. Purification by flash chromatography (Et_2_O/pentane, 85/15) delivered the corresponding HNK-*Bis*-PAc (181 mg, 85% yield). HRMS calculated for HNK-*Bis*-PAc C_24_H_26_O_4_ [MH]^+^ 396.2169, found, 396.2171.

***7.2 NMR Results***

^1^H NMR (400.13 MHz, CDCl_3_), δ 7.27-7.24 (2H, m), 7.21-7.16 (2H, m), 7.08-7.02 (2H, m), 6.04-5.84 (2H, m), 5.16-5.02 (4H, m), 3.43-3.28 (4H, m), 2.61 (2H, q, *J* = 7.6), 2.36 (2H, q, *J* = 7.6), 1.29 (3H, t, (6H, m, *J* = 7.6), 1.07 (3H, t, (6H, m, *J* = 7.6). ^13^C NMR (75 MHz, CDCl_3_) δ 172.9, 172.7, 148.4, 146.1, 138.0, 136.9, 135.8, 135.5, 133.9, 131.6, 130.9, 130.8, 128.6, 127.9, 122.7, 122.2, 116.3, 116.2, 39.6, 34.6, 27.7, 27.6, 9.1, 8.9.

**8. HNK-*Bis*-BAc** (3',5-diallyl-[1,1'-biphenyl]-2,4'-diyl dibutyrate)

***8.1 Synthesis***

To a mixture of HNK (0.15 g, 0.56 mmol) in CH_2_Cl_2_ (4 mL) was added butyryl chloride (200 µL, 1.5 mmol) in the presence of triethylamine (153 µL, 1.15 mmol). The mixture was stirred at room temperature for 2 h. Then, water was added to the mixture and the product was extracted by Et_2_O. The organic layer was dried over Na_2_SO_4_, and the solvent was removed under reduced pressure. Purification by flash chromatography (Et_2_O/pentane, 85/15) delivered the corresponding HNK-*Bis*-BAc (201 mg, 87 % yield), ESI-MS (electrospray ionization mass spectrometry) for HNK-*Bis*-BAc C_26_H_30_O_4_ [MH]^+^ 407.8.

***8.2 NMR Results***

^1^H NMR (400.13 MHz, CDCl_3_), δ 7.28-7.25 (2H, m), 7.21-7.15 (2H, m), 7.08-7.02 (2H, m), 6.04-5.84 (2H, m), 5.16-5.02 (4H, m), 3.43-3.28 (4H, m), 2.57 (2H, t, *J* = 7.3), 2.33 (2H, t, *J* = 7.3), 1.81 (2H, sext., *J* = 7.5, 14.9), 1.60 (2H, sext., *J* = 7.5, 14.9), 1.08 (3H, t, *J* = 7.3), 0.87 (3H, t, *J* = 7.3). ^13^C NMR (75 MHz, CDCl_3_) δ 172.1, 171.9, 148.4, 146.1, 138.0, 136.9, 135.7, 135.5, 134.0, 131.6, 130.9, 128.6, 128.0, 122.8, 122.2, 116.3, 116.2, 39.6, 36.2, 36.0, 34.6, 18.5, 18.2, 13.7, 13.5.

**Supplementary Figures**

Supplementary figures are presented on the subsequent pages.

^1^H NMR (400.13 MHz, CDCl_3_), HNK-Ac

^^

^13^CAPT (75 MHz, CDCl_3_)

^1^H NMR (400.13 MHz, CDCl_3_), HNK-PAc

^13^CAPT (75 MHz, CDCl_3_)

^1^H NMR (400.13 MHz, CDCl_3_), HNK-BAc

^13^CAPT (75 MHz, CDCl_3_)

^1^H NMR (400.13 MHz, CDCl_3_), HNK-HAc

^13^CAPT (75 MHz, CDCl_3_)

**Figure S1. NMR results for HNK-Ac, HNK-PAc, HNK-BAc, and HNK-HAc.**

^1^H NMR (400.13 MHz, CDCl_3_), HNK-*Bis*-Ac

^^

^13^CAPT (75 MHz, CDCl_3_)

^1^H NMR (400.13 MHz, CDCl_3_), HNK-*Bis*-PAc

^13^CAPT (75 MHz, CDCl_3_)

^1^H NMR (400.13 MHz, CDCl_3_), HNK-*Bis*-BAc

**Figure S2. NMR results for HNK-*Bis*-BAc and HNK-*Bis*-PAc.**

**
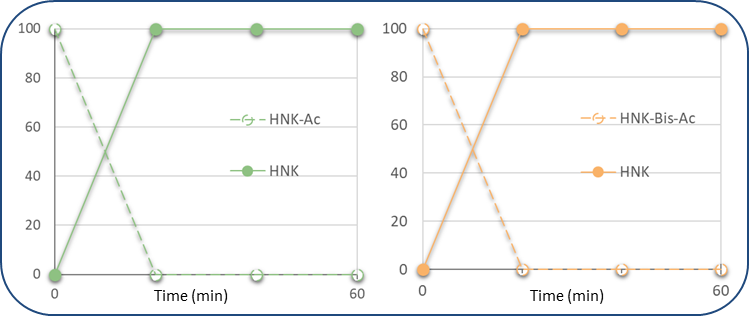
**

**Figure S3. Enzymatic hydrolysis of HNK-Ac and HNK-*Bis*-Ac.**


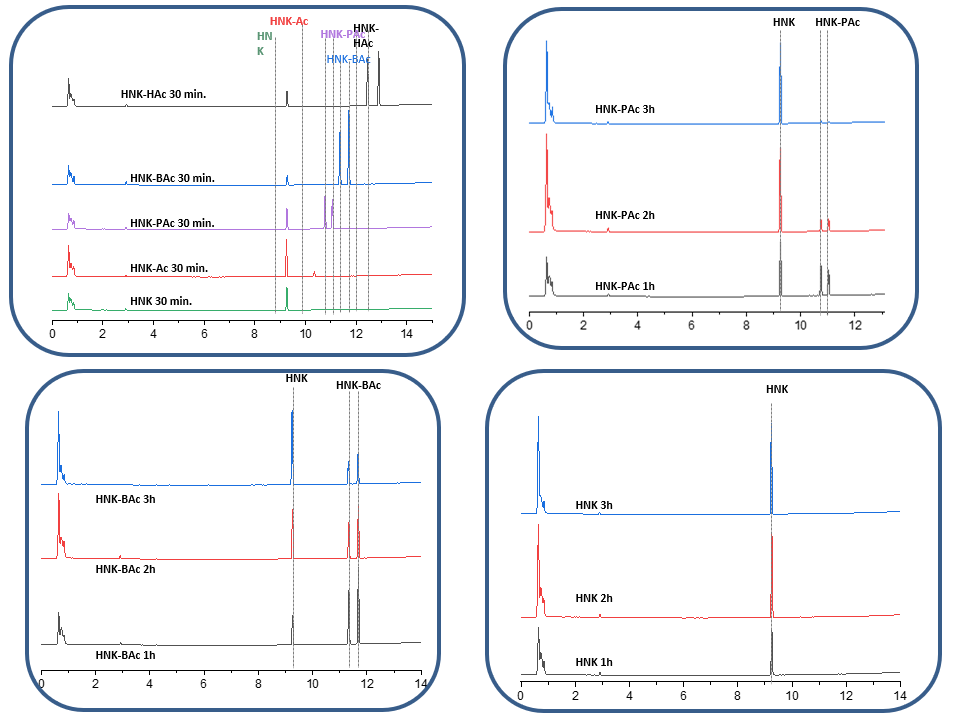


**Figure S4. Uptake of HNK-SCFA esters.**

**
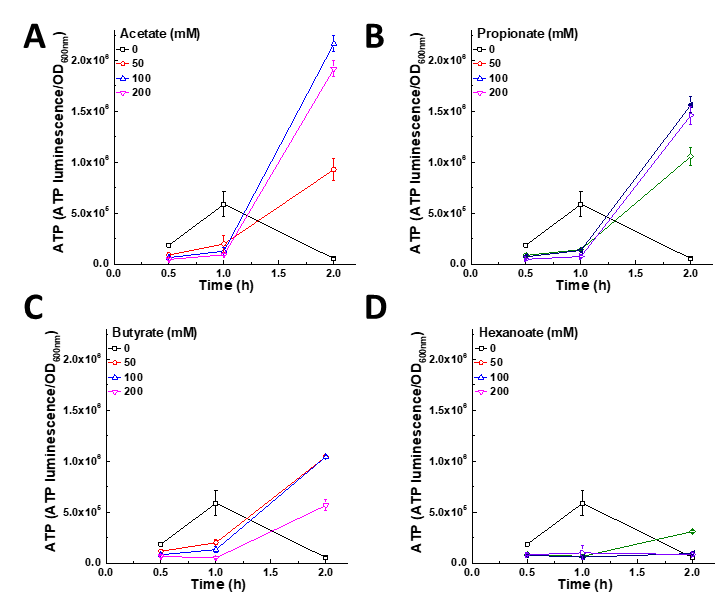
**

**Figure S5. Effects of SCFAs on the ATP level.** *E. faecalis* was treated with acetate **(A)**, propionate **(B)**, butyrate **(C)**, and hexanoate **(D)** as indicated for 0.5 h, 1 h, and 2 h.

**Figure S6. Effects of HNK analogs on the bacterial cytotoxicity.** *E. faecalis* was treated with HNK analogs as indicated for 3 h, and cell death was monitored in real time by Sytox Green staining. Data shown are the means ± SD for n=4.

**Figure S7. Chemical structures of butyrate derivatives.**

**Table S1. Calculated values of the octanol/water partition coefficients**

**
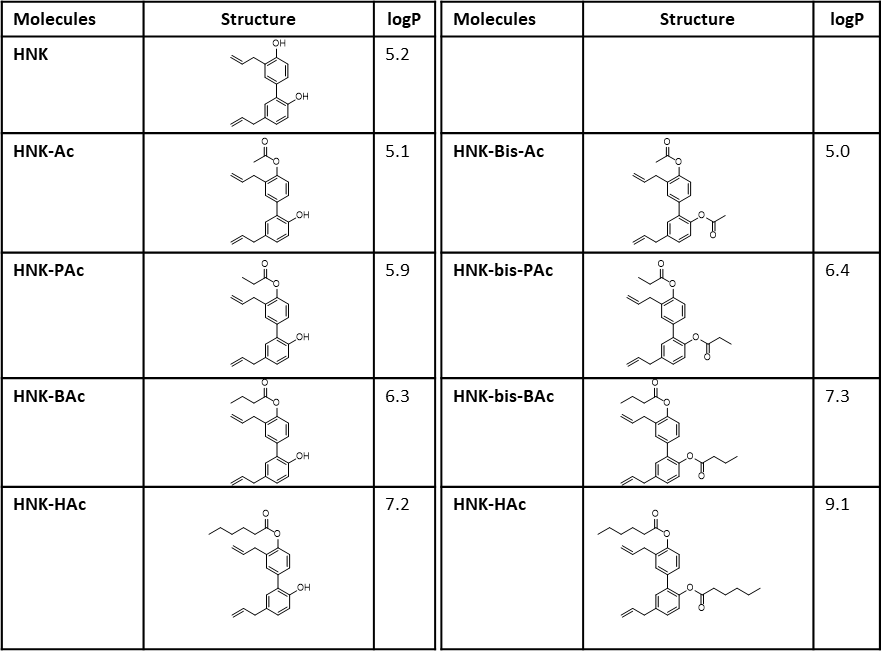
**
